# Cerebral Metastasis of a Gastrointestinal Stromal Tumor: A Case Report and Literature Review

**DOI:** 10.7759/cureus.85857

**Published:** 2025-06-12

**Authors:** Diogo D Lopes, Renato Pereira, Elisabete Couto, Diana Freitas, Ana Daniela Marques

**Affiliations:** 1 Medical Oncology, Unidade Local de Saúde de Braga, Braga, PRT; 2 Neurosurgery, Unidade Local de Saúde de Braga, Braga, PRT

**Keywords:** cerebral metastasis, gastrointestinal oncology, gastrointestinal stromal tumor (gist), imatinib and sunitinib, rare cancer

## Abstract

Gastrointestinal stromal tumors (GISTs) are rare mesenchymal neoplasms originating from the gastrointestinal tract. These tumors frequently harbor activating mutations in the receptor tyrosine kinase (KIT) or in the platelet-derived growth factor receptor alpha (PDGFRA) gene, which guide treatment with tyrosine kinase inhibitors (TKIs). Although GISTs commonly metastasize to the liver and peritoneum, involvement of the central nervous system (CNS) is exceptionally uncommon.

We report a case of a 58-year-old male with metastatic gastric GIST, treated with imatinib, followed by sunitinib and regorafenib, between October 2021 and December 2023, when he presented with sudden visual disturbances and a transient loss of consciousness. Imaging revealed a large extra-axial lesion in the right frontal region adjacent to a lytic bone lesion. Craniotomy and histopathology confirmed cerebral metastasis from GIST. The patient underwent cranial radiotherapy and was proposed for treatment with ripretinib, but treatment was not initiated due to clinical deterioration. He passed away in March 2024.

CNS metastasis from GISTs is a rare phenomenon. Most cases occur in the context of advanced disease and carry a poor prognosis. This case underscores the absence of effective systemic treatments for CNS disease, as most TKIs, including imatinib and ripretinib, have poor CNS penetration. Surgical resection and radiotherapy provide symptomatic relief but are not curative. This case highlights the need for novel therapeutic approaches targeting CNS metastases and further exploration of molecular mechanisms that enable atypical metastatic spread.

This report contributes to the sparse literature on CNS involvement in GIST, emphasizing the need for a multidisciplinary approach and the development of therapies that can penetrate the blood-brain barrier (BBB) to improve outcomes in patients with advanced GIST.

## Introduction

Gastrointestinal stromal tumors (GISTs) are rare mesenchymal neoplasms, accounting for 1-2% of all gastrointestinal (GI) malignancies. Most commonly arising in the stomach (60%), these tumors are characterized by activating mutations in the receptor tyrosine kinase (KIT) proto-oncogene (approximately 75-80% of cases) and the platelet-derived growth factor receptor alpha (PDGFRA) gene (5-10%). These mutations drive uncontrolled cell proliferation, providing actionable targets for tyrosine kinase inhibitors (TKIs) such as imatinib, which has significantly improved outcomes in patients with advanced or metastatic GIST [1,2].

Metastases in GISTs typically involve the liver and peritoneum, with central nervous system (CNS) involvement being exceptionally rare. The blood-brain barrier (BBB) may account for the limited CNS spread of GISTs, but it also poses a challenge for treatment given the limited penetration of many TKIs across the BBB [3]. This report presents a unique case of cerebral metastasis from a gastric GIST, contributing to the limited literature on this phenomenon and exploring therapeutic challenges and potential genetic underpinnings.

## Case presentation

A 58-year-old male patient with a history of alcoholism and essential hypertension presented to the Emergency Department in September 2021 with a four-month history of asthenia and right hypochondrial pain radiating to the lumbar region. Initial investigations revealed microcytic, hypochromic anemia (hemoglobin 8 mg/dL; normal range: 13.0-17.0 mg/dL) and a marked thickening of the gastric fundus wall, along with multiple liver lesions on computed tomography (CT) scan, suggestive of metastasis. Upper gastrointestinal endoscopy showed an infiltrative, ulcerated lesion in the gastric fundus, whose histopathological analysis confirmed a GIST (positive for DOG1, CD117, and CD34 on immunohistochemistry). Due to worsening anemia (hemoglobin 7.4 mg/dL; normal range: 13.0-17.0 mg/dL), he underwent gastric hemostatic radiotherapy (20 Gray in five fractions).

**Figure 1:**
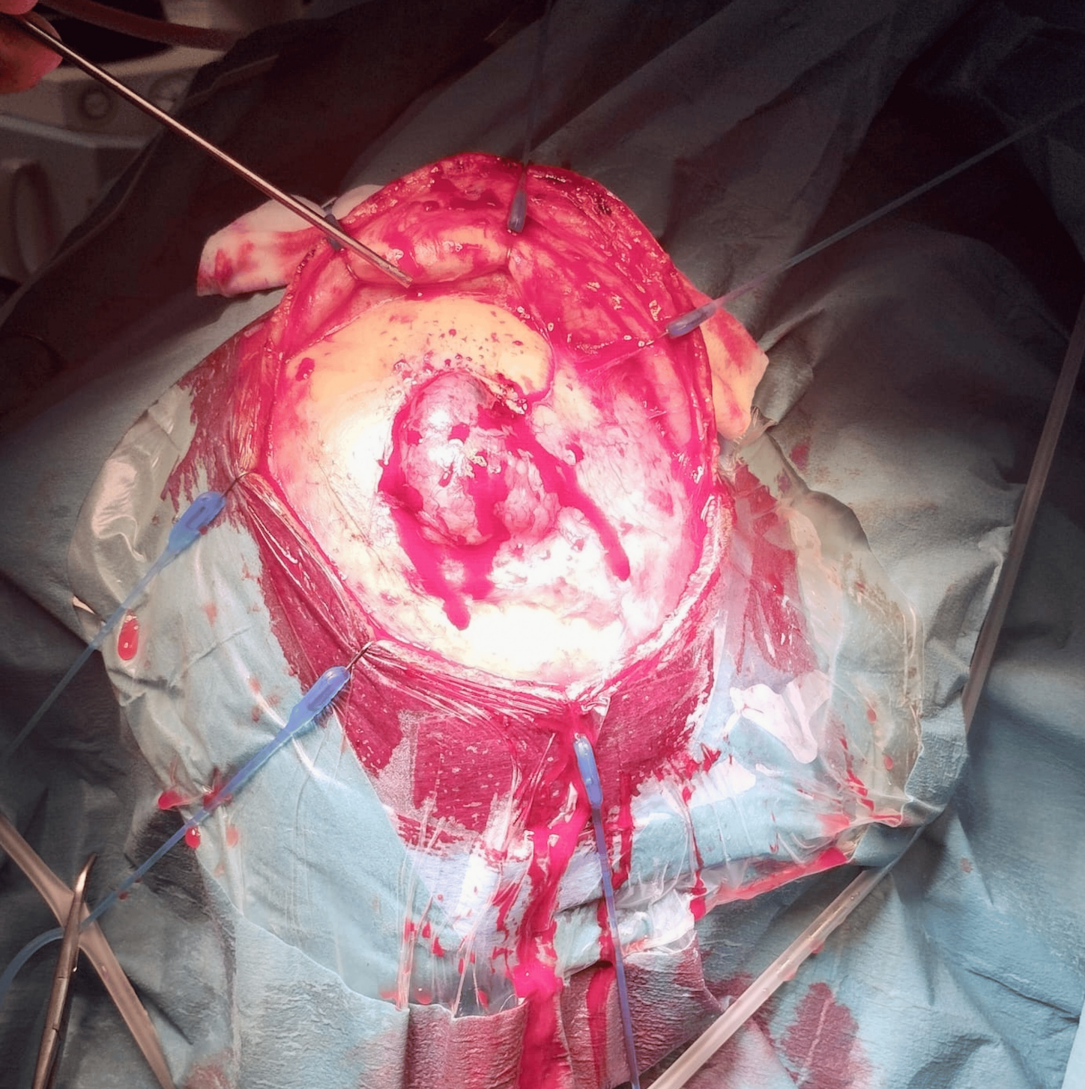
An intraoperative image showing the extra-cranial extension of tumor after the skin flap was raised.

**Figure 2:**
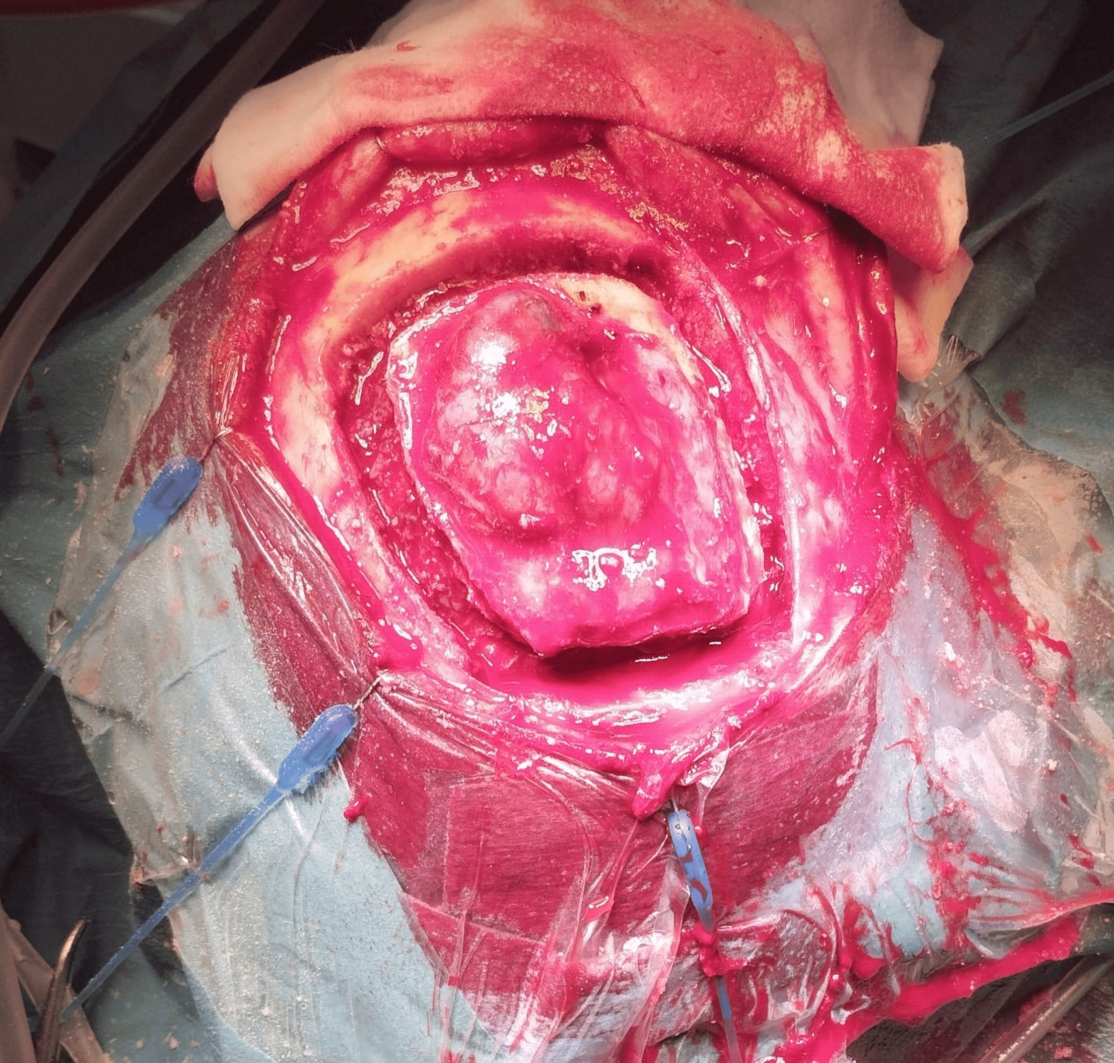
An intraoperative image showing the extra-cranial tumor extension after the bone flap was raised.

After multidisciplinary discussion, the patient began treatment with imatinib (400 mg/day) in October 2021. Due to hepatic disease progression in October 2022, the treatment was switched to sunitinib (50 mg/day for four weeks followed by a two-week break) until July 2023, when further hepatic disease progression led to third-line palliative treatment with regorafenib (160 mg/day for three weeks followed by a one-week break). In December 2023, the patient developed sudden visual disturbances and an episode of loss of consciousness. A brain CT scan revealed a large extra-axial lesion in the right frontal region adjacent to a lytic bone lesion, suggestive of metastatic disease. Due to the rarity of GIST metastasis to the CNS and the need for biopsy confirmation, a right frontal craniotomy with excision of the calvarial lesion and cranioplasty was performed in January 2024, confirming cerebral metastasis from gastric GIST (Figures 1, 2). Cranioplasty was performed using a titanium mesh, and the underlying dura mater was found to be intact after lesion removal.

As new calvarial bone lesions appeared, treatment with radiotherapy was proposed, followed by fourth-line palliative treatment with ripretinib. Following radiotherapy, the patient’s condition deteriorated rapidly, and he passed away in March 2024 before initiating ripretinib.

## Discussion

Cerebral metastasis from GISTs is exceptionally rare, with few cases reported in the literature. GISTs spread predominantly to the liver and peritoneum, with the CNS remaining an uncommon site. This rarity may be due to the protective nature of the BBB, which limits the dissemination of tumor cells to the CNS [3]. However, as demonstrated in this case, cerebral metastasis can occur in patients with progressive disease despite multiple lines of systemic therapy, raising important questions about the mechanisms driving atypical metastatic spread and the limitations of current treatment options.

Genetic mutations play a leading role in the pathophysiology of GISTs, influencing both tumor behavior and therapeutic responses. Approximately 75-80% of GISTs harbor mutations in the KIT proto-oncogene, specifically in exons 11, 9, 13, and 17, with exon 11 mutations being the most common [1]. These mutations lead to constitutive activation of the KIT receptor, driving uncontrolled cell proliferation. Another 5-10% of GISTs exhibit mutations in the PDGFRA gene, often seen in tumors located in the stomach [2]. Such mutations have become actionable targets with the advent of TKIs like imatinib, which has significantly improved outcomes for patients with advanced or metastatic GIST [1]. However, secondary resistance to imatinib frequently develops due to additional mutations in the KIT or PDGFRA genes, requiring the use of second- and third-line therapies, such as sunitinib and regorafenib [4].

Despite these advances, the efficacy of TKIs in controlling GIST metastases outside typical sites remains limited [5]. The present case illustrates the challenges faced when GIST spreads to the CNS, where the BBB restricts TKI penetration, reducing drug efficacy. The progression observed in this patient, who sequentially received imatinib, sunitinib, and regorafenib, reflects the limitations of available TKIs in controlling aggressive disease with atypical metastatic behavior. Interestingly, ripretinib, a fourth-line TKI approved for advanced GIST, also has limited CNS penetration, underscoring an ongoing lack of therapeutic options for CNS metastases [6].

A review of other reported cases highlights the therapeutic challenges and poor prognosis associated with CNS metastasis in GIST. Most documented cases report neurological symptoms such as headache, visual alterations, or seizures as the initial presentation, similar to the symptoms noted in our patient [7]. In these cases, surgical resection followed by stereotactic radiosurgery or whole-brain radiotherapy has been the mainstay of treatment. While surgery and radiotherapy may provide symptomatic relief and local control, they are rarely curative, as CNS metastases are often indicative of advanced disease with limited systemic options. Survival in these patients remains poor, with most dying due to disease progression within months of CNS involvement.

This case raises several questions regarding the potential molecular mechanisms that could drive unusual metastatic patterns in GIST. It is plausible that alterations beyond KIT and PDGFRA mutations may contribute to CNS metastasis, as some studies have suggested that mutations in genes involved in epithelial-mesenchymal transition and the PI3K/AKT/mTOR signaling pathway could enhance tumor invasiveness and facilitate BBB penetration [8]. However, these pathways remain underexplored in GIST, particularly in cases with atypical metastatic spread. Further research into these molecular drivers is warranted, as a deeper understanding could reveal new therapeutic targets and predictive markers for CNS metastases.

The therapeutic landscape for GIST with CNS metastasis remains limited, partly due to the rarity of the condition and the lack of BBB-penetrating agents [3]. While TKIs such as cabozantinib have shown improved BBB penetration in other malignancies, their efficacy in GIST has not been thoroughly studied, especially in the context of CNS disease [9]. Immunotherapy and targeted agents directed at the PI3K/AKT/mTOR pathway have shown promise in preclinical studies and could represent future treatment options if supported by clinical trials. Moreover, as liquid biopsy and next-generation sequencing become more prevalent, these tools may facilitate early detection of mutations associated with aggressive or atypical metastatic patterns, enabling preemptive therapeutic strategies [10].

This case underscores the importance of a multidisciplinary approach in managing patients with GIST, particularly those with rare metastatic presentations. The collaboration between oncologists, radiologists, neurosurgeons, and radiation oncologists is crucial to provide comprehensive care that addresses both systemic and CNS disease. As demonstrated in this case, surgical resection of CNS lesions may be essential for diagnostic confirmation and symptom management, while radiotherapy provides local control when systemic therapies are inadequate. However, the limited efficacy of current systemic treatments in the CNS suggests a need for novel therapies with enhanced CNS activity. Developing agents that can effectively cross the BBB and target both primary and secondary resistance mechanisms will be essential in improving outcomes for patients with advanced GIST and CNS metastasis.

## Conclusions

This case contributes to the limited literature on cerebral metastasis in GISTs, underscoring the complexity and rarity of CNS involvement in these patients. Although targeted therapies like imatinib, sunitinib, regorafenib, and ripretinib have transformed the management of metastatic GIST, their limited ability to cross the BBB presents a challenge for treating CNS metastases. Current approaches often rely on palliative measures, including surgery and radiotherapy, which may provide symptom relief but do not substantially change prognosis, highlighting the need for discussion of these cases in a multidisciplinary team meeting.

The atypical presentation in this patient raises important questions about alternative molecular mechanisms that may predispose GISTs to unusual metastatic patterns. Further research into these mechanisms could support improved risk stratification and targeted interventions for high-risk patients. Developing TKIs with better CNS penetration, exploring alternative therapeutic pathways, and including patients with CNS metastasis in clinical trials could lead to better clinical outcomes. For now, cases like this highlight the lack of effective treatment options and the need for innovative approaches to improve both survival and quality of life for this rare patient population.
